# Supplementary material for: Enzalutamide Prior to Radium-223 Is Associated with Better Overall Survival in Metastatic Castration-Resistant Prostate Cancer Patients Compared to Abiraterone—A Retrospective Study
Source: Cancers (Basel). 2023 Jul 6;15(13):3516. doi: 10.3390/cancers15133516 (PMC10340515; doi:10.3390/cancers15133516)
Supplement: Supplementary file 1 [file cancers-15-03516-s001.zip › supplementary Figure S1.pdf]

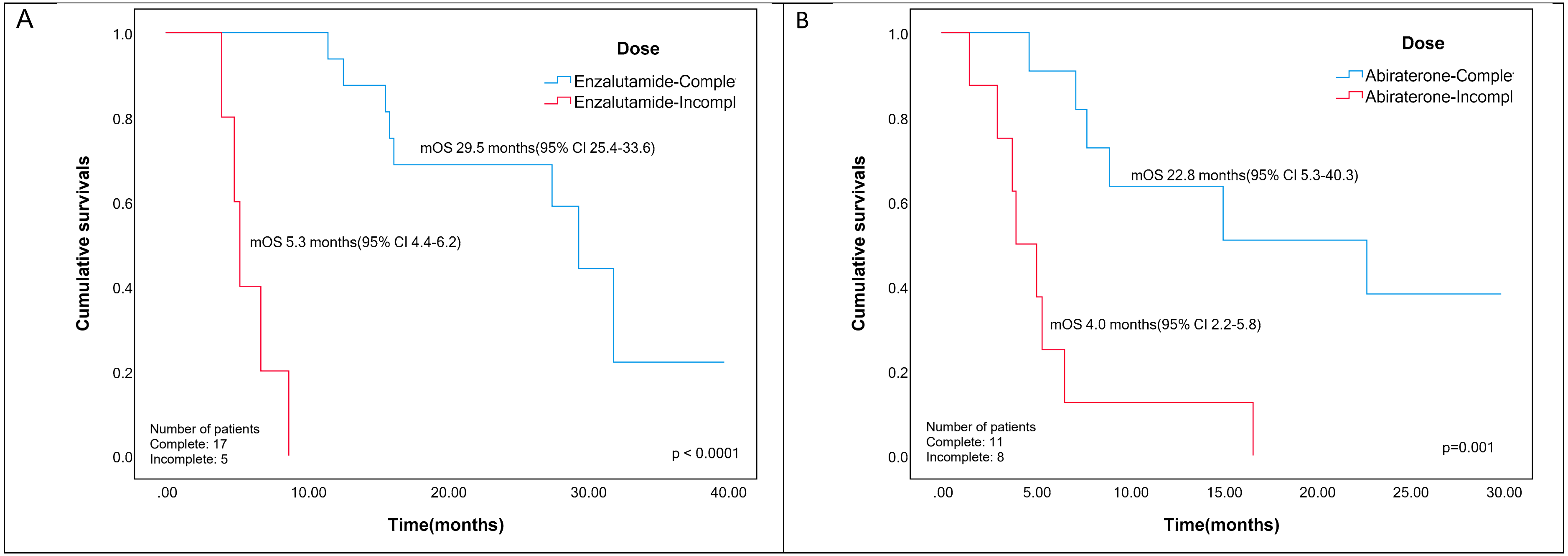


Supplementary Figure S1: The Kaplan-Meier curve illustrates the (A) comparison between patients in the enzalutamide group who received complete radium-223 treatment (≥5 doses) versus those with incomplete treatment, and (B) comparison between patients in the Abiraterone group who received complete radium-223 treatment (≥5 doses) versus those with incomplete treatment.
