# Supplementary material for: Enzalutamide Prior to Radium-223 Is Associated with Better Overall Survival in Metastatic Castration-Resistant Prostate Cancer Patients Compared to Abiraterone—A Retrospective Study
Source: Cancers (Basel). 2023 Jul 6;15(13):3516. doi: 10.3390/cancers15133516 (PMC10340515; doi:10.3390/cancers15133516)
Supplement: Supplementary file 1 [file cancers-15-03516-s001.zip › supplementary Table S1.pdf]

Supplementary Table S1: Case details of all patients with skeletal-related events.

Case	NHA	ALK-p (U/L)	ALK-p decrease	PSA (ng/mL)	Dose of Rad-223	Extent of disease	Time to first SRE (months)	Event	Management
1	Enzalutamide	64	Yes	76.1	6	>20 metastases	9.8	Spinal cord compression by metastatic tumor	Surgery
2	Enzalutamide	80	No	31.15	6	6-20 metastases	7.2	Pelvic bone pain	IMRT
3	Enzalutamide	98	No	11.13	5	6-20 metastases	12.1	Right ilium fracture	Surgery
4	Abiraterone+ prednisolone	51	No	20.29	6	<6 metastases	11.4	Left intertrochanteric fracture	Surgery
5	Abiraterone+ prednisolone	58	No	29.27	6	6-20 metastases	7.1	Right femoral head and intertrochanteric fracture	Surgery

All patients had completed radium-223. Only one patient had ALK-p decrease after radium-223 administration.

Abbreviation: NHA: novel hormonal agents, ALK-p: alkaline phosphatase, SRE: skeletal-related event, IMRT: Intensity-modulated radiation therapy
